# Socioeconomic and geographic inequalities in antenatal and postnatal care components in India, 2016–2021

**DOI:** 10.1038/s41598-024-59981-w

**Published:** 2024-05-03

**Authors:** Hyejun Chi, Sohee Jung, S. V. Subramanian, Rockli Kim

**Affiliations:** 1 Interdisciplinary Program in Precision Public Health, Department of Public Health Sciences, Graduate School of Korea University, 145 Anam-Ro, Seongbuk-Gu, Seoul, 02841 Republic of Korea; 2 Harvard Center for Population and Development Studies, 9 Bow Street, Cambridge, MA 02138 USA; 3 Department of Social and Behavioral Sciences, Harvard T.H. Chan School of Public Health, 677 Huntington Ave, Boston, MA 02115 USA; 4 Division of Health Policy and Management, College of Health Sciences, Korea University, 145 Anam-ro, Seongbuk-Gu, Seoul, 02841 Republic of Korea

**Keywords:** Public health, Epidemiology

## Abstract

Despite the well-known importance of high-quality care before and after delivery, not every mother and newborn in India receive appropriate antenatal and postnatal care (ANC/PNC). Using India’s National Family Health Surveys (2015–2016 and 2019–2021), we quantified the socioeconomic and geographic inequalities in the utilization of ANC/PNC among women aged 15–49 years and their newborns (N = 161,225 in 2016; N = 150,611 in 2021). For each of the eighteen ANC/PNC components, we assessed absolute and relative inequalities by household wealth (poorest vs. richest), maternal education (no education vs. higher than secondary), and type of place of residence (rural vs. urban) and evaluated state-level heterogeneity. In 2021, the national prevalence of ANC/PNC components ranged from 19.8% for 8 + ANC visits to 91.6% for maternal weight measurement. Absolute inequalities were greatest for ultrasound test (33.3%-points by wealth, 30.3%-points by education) and 8 + ANC visits (13.2%-points by residence). Relative inequalities were greatest for 8 + ANC visits (1.8 ~ 4.4 times). All inequalities declined over time. State-specific estimates were overall consistent with national results. Socioeconomic and geographic inequalities in ANC/PNC varied significantly across components and by states. To optimize maternal and newborn health in India, future interventions should aim to achieve universal coverage of all ANC/PNC components.

## Introduction

Maternal mortality ratio in India was 97 deaths per 100,000 live births in 2018–2020, which is progressively approaching what the Sustainable Development Goals envision to achieve by 2030 (i.e., less than 70 deaths per 100,000 live births)^1,2^. However, pregnancy-related complications continue to be the leading causes of deaths among Indian girls aged 15–19 years, indicating the need to intervene on maternal health services in the context of India^3^. Moreover, although high-quality care provided by skilled health professionals during pregnancy (antenatal care; ANC) as well as during and after delivery (postnatal care; PNC) can ensure the best health conditions for both mothers and their newborns, not every mother and newborn in India receive appropriate ANC/PNC^4–6^. In 2019–2021, less than 60% of Indian mothers who gave birth in the last five years made a minimum four ANC visits and 20% of newborns failed to receive PNC from health professionals within two days of birth^6,7^.

Prior literature identified a diverse range of demographic and socioeconomic characteristics, such as maternal age and educational level, social groups, marital status and parity, place of residence, media exposure, women’s autonomy, and knowledge of pregnancy to be associated with mother’s utilization of ANC/PNC in India^8–15^. Specifically, mothers with higher socioeconomic status (SES) were found to have more complete ANC/PNC given the availability of resources for healthcare expenses and greater access to health information that allow them to assign higher priority to healthcare use^8,10,11,14^. Maternal education is also known to improve mother’s communication skills with other household members as well as her decision-making ability over health issues^8,10,14^. Higher SES can affect mother’s awareness of healthcare and its benefit through greater exposure to mass media as well, which is critical for changes in health behaviors^10,11^.

Regional variation in the use of ANC/PNC is also substantial, with mothers in urbans areas and southern regions of India being more likely to use ANC/PNC compared to others. This can be explained partly by poor accessibility to health services in rural areas as well as concerns regarding the quality of health services including absence and low skill level of health professionals and lack of medicine^9,10,12,13^. Moreover, regional disparities in overall development in terms of economy, education, and health infrastructure may be responsible for differential use of maternity care between mothers in southern states including Andhra Pradesh, Kerala and Tamil Nadu, and the rest of the country^8,11,13,14^. Given the decentralized health system in India wherein most health policies and programs are planned and implemented at the state-level, such discrepancies across states have been exacerbated over time^16,17^. Hence, it is necessary to quantify the extent of socioeconomic and geographic inequality in the use of maternity care and monitor the change over time so as to identify priority groups in India.

What should be further considered in line with the utilization of ANC/PNC, which is typically assessed by the number and timing of care visits and the type of care providers, is the kind of health services that were specifically provided during the uptake of care. Proper counselling, health education, physical diagnosis, and distribution of nutritional supplements are essential components that mothers and newborns should receive as part of their ANC/PNC^6^. Investigating whether these individual components were properly provided can describe the comprehensive completion of care. Promoting the comprehensive set of ANC/PNC is important to minimize the health burden of Indian mothers and newborns given that poor quality care can cause excessive maternal and neonatal deaths than non-utilization of care^18^. Nonetheless, to the best of our knowledge, only limited attempt has been made to analyze the association between individual components of ANC/PNC and sociodemographic and geographic predictors, and the data employed in these studies are now outdated^19–22^.

Thus, the present study aims to (a) identify all relevant ANC/PNC components and assess their changes over time (2015–2016 to 2019–2021) in the utilization rate, (b) quantify the absolute and relative inequality in each by socioeconomic status (wealth and education) and geographic predictor (rural versus urban), and (c) evaluate heterogeneity by states, using the latest nationally representative data from India.

## Methods

### Data and study sample

The Demographic and Health Surveys (DHS) are cross-sectional surveys that are known for standardized and nationally representative sampling of participants and collection of a wide range of monitoring and impact evaluation indicators for population, health, and nutrition^23^. National Family Health Surveys (NFHS) are equivalent to DHS in India. For this study, two cross-sectional surveys from 2015–2016 (NFHS-4) and 2019–2021 (NFHS-5) were used.

NFHS-5 collected data on 724,115 women aged 15–49 years across 707 districts and 36 states and union territories of India. Our analysis was restricted to mothers and their latest birth in the last 5 years preceding the survey. From 232,920 mothers who met the delivery criterion, a total of 150,611 mothers and their newborns were included in the final analysis after excluding those who were missing data on ANC/PNC components and other covariates (Supplementary Fig. S1a online). For NFHS-4, the same inclusion procedure was applied, and this left a total of 161,225 mothers and newborns (Supplementary Fig. S1b online).

### Predictor variables

To evaluate the socioeconomic and geographic inequalities in the utilization of ANC/PNC, household wealth index, maternal education level, and type of place of residence were selected as the primary predictor variables. Household wealth index score, a composite index of relative standard of living, was created in DHS using principal component analyses of household characteristics and assets, which was then divided into quintiles (poorest, poorer, middle, richer, richest)^23^. This measure has been validated in prior studies^24^. In terms of reporting inequality by household wealth, the comparison was made between the poorest and the richest quintiles. Education level was categorized as no education (0 year), primary (1–5 years), secondary (6–12 years), and higher than secondary education (13 years and more). Similar to household wealth, inequality by education level was calculated by comparing mothers with no education versus higher than secondary education. Type of place of residence was classified as urban versus rural residence, wherein rural residence served as a reference.

### Outcome variables

12 and six individual components of ANC and PNC, respectively, were selected based on the World Health Organization’s (WHO) recommendations and other previous literature (Table 1)^6,19–21,25–27^. For ANC components, we used number of ANC visits (eight or more visits), timing of first ANC visit (within 12 weeks of pregnancy), type of care provider (health professionals including doctors, nurses, auxiliary nurse midwives, lady health visitors, and other health personnel), and whether following services were provided: measurement of weight and blood pressure, collection of blood and urine sample, counselling on pregnancy complications and where to go for complications, ultrasound test, receipt of iron/folic supplements, and tetanus toxoid containing vaccination (TT-CV). PNC components included timing of both mother and newborn’s checkup (within 24 h of delivery), provider of the checkup (health professionals), weigh measurement for a newborn, and timing of breastfeeding initiation (within an hour of delivery). Each component was coded as 1 (yes) if a mother or a newborn satisfied its suggested definition and 0 (no) otherwise.

**Table 1:** List and definition of 18 individual components of antenatal and postnatal care.

Component	Definition	References
Antenatal care (12)		
Number of visits	A mother made ≥ 8 antenatal care (ANC) visits	^6^
Timing of first visit	A mother made her first ANC visit in the first trimester of pregnancy (≤ 12 weeks)	^6,25^
Provider	A mother saw health professionals* for ANC	^25^
Weight measurement	A mother was weighed at least once as part of ANC	^6,25^
Blood pressure measurement	A mother’s blood pressure was measured at least once as part of ANC	^6,25^
Urine sample collection	A mother gave her urine sample at least once as part of ANC	^6,25^
Blood sample collection	A mother gave her blood sample at least once as part of ANC	^6,25^
Counselling on pregnancy complications	A mother was told about at least one of the signs of pregnancy complications during ANC visits	^6,25^
Counselling on where to go for complications	A mother was told where to go if she had any pregnancy complications during ANC visits	^6,25^
Ultrasound test	A mother had an ultrasound test at least once as part of ANC	^6^
Receipt of iron supplement	A mother was given or bought any iron folic acid tablets/syrup during pregnancy	^6,25^
Tetanus vaccination	If a mother has not previously been vaccinated, received ≥ 2 doses of tetanus toxoid containing vaccine (TT-CV) during pregnancy. Otherwise, received one dose if TT-CV during pregnancy	^6,25^
Postnatal care (6)		
Timing of first check for mother	A mother received postnatal check within 24 h of delivery	^24^
Timing of first check for newborn	A newborn received postnatal check within 24 h of delivery	^24^
Provider for mother	Health professionals checked mother’s health after delivery	
Provider for newborn	Health professionals checked newborn’s health after delivery	
Weight measurement for newborn	A mother reported that her newborn was weighed at birth with specific weight in kilograms (0.5–9.0kg)	^24^
Initiation of breastfeeding	A newborn was put to his/her mother’s breast within an hour of delivery	^26^

*Health professionals include doctors, nurses, auxiliary nurse midwives, midwives, lady health visitors and other health personnel.

### Other covariates

Covariates that represent other demographic and socioeconomic features of mothers and newborns were adjusted for in multivariable analyses and these included: (a) maternal age at childbirth (< 20, 20–24, 25–29, 30–34, 35–39, 40–44, 45–49 years); (b) sex of newborn (boy, girl); (c) birth order of newborn (1, 2–3, 4+); (d) maternal marital status (currently married/living with a partner, never in a union, divorced/widowed/separated); (e) caste/tribe (scheduled caste/tribe including other backward classes, others); and (f) religion (Hindu, Muslim, Christian, others).

### Statistical analyses

We first examined the national prevalence of mothers and newborns who received each component of ANC/PNC in the study sample of 2016 and 2021, separately. Change over time in socioeconomic and geographic inequalities in the prevalence for each component were reported in absolute and relative terms. For absolute inequality (AI), we subtracted the prevalence of the comparing group (e.g., the richest quintile) from that of the reference group (e.g., the poorest quintile). Relative inequality (RI) was calculated via dividing the prevalence from the counterpart by that from the reference group. Absolute and relative inequalities were assessed in tandem since it is important to consider both measures when assessing inequality in a single-population case^28^. Either absolute or relative inequality measure alone fails to show the overall health of the target population, as well as how consistent and large the difference is going to be on the other scale.

To evaluate the heterogeneity in absolute and relative inequalities in ANC/PNC components across states, stratified analysis was performed using the latest survey (NFHS-5). States with sample size smaller than 500 were excluded from the analysis. As a result, Andaman and Nicobar Islands (N = 314), Chandigarh (N = 131), Goa (N = 192), Ladakh (N = 245), Lakshadweep (N = 203), and Sikkim (N = 433) were excluded.

Multivariable logistic regression models were conducted to identify socioeconomic and geographic inequalities in the receipt of ANC/PNC components after adjusting for other relevant covariates. We adjusted for demographic and socioeconomic features of mothers and newborns (maternal age at birth, sex and birth order of newborn, maternal marital status, caste/tribe, religion) in a separate model for each ANC/PNC component.

Statistical outputs obtained from the logistic regression were reported in odds ratio (OR) with 95% confidence intervals (CI) and were considered statistically significant at p-value < 0.05. All descriptive analyses were weighted, and logistic models adjusted for clustered standard errors given the complex survey design. All statistical analyses were performed via STATA/MP 16.1^29^.

## Results

Overall, most mothers were from rural areas (74.6–78.5%) and had secondary or higher education (58.0–67.9%) (Supplementary Table S1 online). The majority of study sample was in their twenties (72.5–73.1%) and currently married or living with a partner (98.3–98.4%).

### Utilization of ANC/PNC components in 2016 vs. 2021

In 2021, all ANC components were completed by majority of mothers, except for making a minimum eight of visits (19.80%) (Table 2). 70% of mothers made their first ANC visit in the first trimester (70.93%) and most visits were provided by health professionals (85.73%). Physical examinations (i.e., measurement of weight and blood pressure, collection of blood and urine sample) were provided for around 90% of mothers. However, the prevalence of receiving proper counselling was relatively low (72.88–76.33%). For preventive measures of ANC, the prevalence ranged from 85.91% for ultrasound test to 91.10% for TT-CV. Most PNC components were provided to mothers and newborns in 2021, except for initiation of breastfeeding within an hour of delivery (43.69%). Majority of mothers (77.07%) and newborns (77.70%) received their first postnatal check within a day of delivery, and these checkups were provided by health professionals (80.00% for mothers, 82.27% for newborns). The completion of weight measurement for a newborn was the highest among all ANC/PNC components (93.96%).

**Table 2:** National prevalence of antenatal and postnatal care components, 2015–2016 (N = 161,225) vs. 2019–2021 (N = 150,611).

	2015–2016 (N = 161,225)	2019–2021 (N = 150,611)
	N (%)*	
Antenatal care		
Number of visits (≥ 8 visits)	33,097 (20.53)	29,816 (19.80)
Timing of first visit (≤ 12 weeks)	95,859 (59.46)	106,833 (70.93)
Provider (health professionals)	128,797 (79.89)	129,119 (85.73)
Weight measurement	122,910 (76.24)	137,905 (91.56)
Blood pressure measurement	121,327 (75.25)	136,756 (90.80)
Urine sample collection	119,456 (74.09)	133,078 (88.36)
Blood sample collection	118,526 (73.52)	133,939 (88.93)
Counselling on pregnancy complications	87,070 (54.01)	109,765 (72.88)
Counselling on where to go for complications	92,283 (57.24)	114,967 (76.33)
Ultrasound test	111,265 (69.01)	129,393 (85.91)
Receipt of iron supplement	126,993 (78.77)	132,759 (88.15)
Tetanus vaccination	142,467 (88.37)	137,202 (91.10)
Postnatal care		
Timing of first check for mother (≤ 24 h)^†^	96,457 (59.83)	116,075 (77.07)
Timing of first check for newborn (≤ 24 h)^†^	37,944 (23.53)	117,020 (77.70)
Provider for mother (health professionals)^†^	105,903 (65.69)	120,486 (80.00)
Provider for newborn (health professionals)^†^	50,767 (31.49)	123,904 (82.27)
Weight measurement for newborn	134,313 (83.31)	141,509 (93.96)
Initiation of breastfeeding (≤ 1 h)	70,409 (43.67)	65,805 (43.69)

*For each component, national prevalence was weighted using mother’s individual sampling weight divided by 1,000,000.

^†^The operationalization of four postnatal care components (timing of first check for mother and newborn, provider for mother and newborn) was done differently in NHFS-4 in 2015–2016 and NFHS-5 in 2019–2021 because a different set of variables was given in each data and thus these components lack comparability between the two time periods.

When assessing change over time between 2016 and 2021, the prevalence of all ANC/PNC components increased (Table 2). The improvement was largest in timing (23.53% to 77.70%) and provider of PNC checkup (31.49% to 82.27%) for newborns. On the other hand, the prevalence of number of ANC visits (~ 20%) and initiation of breastfeeding (~ 43%) remained stable.

### Socioeconomic and geographic inequalities in utilization of ANC/PNC components

#### Household wealth

**Figure 1:**
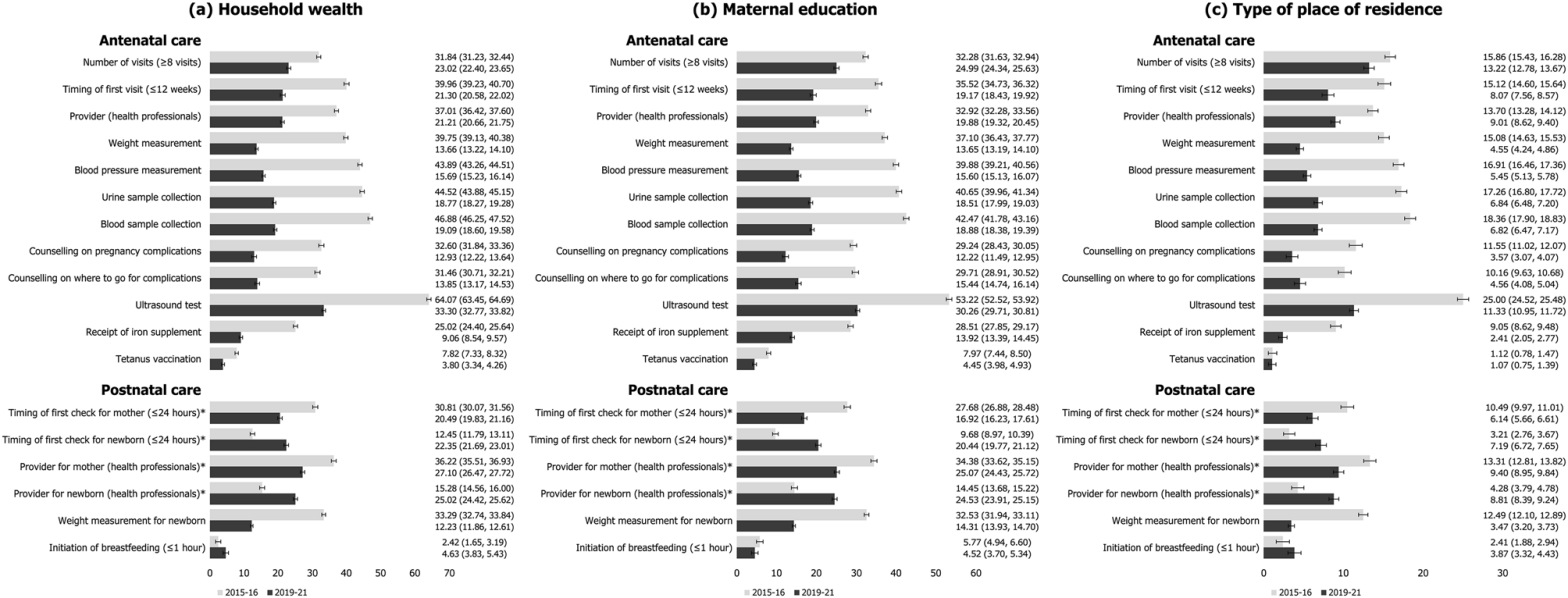
Change in absolute inequalities in the prevalence of antenatal and postnatal care components (%-point) stratified by socioeconomic and geographic predictors, 2015–2016 (N = 161,225) vs. 2019–2021 (N = 150,611). *The operationalization of four postnatal care components (timing of first check for mother and newborn, provider for mother and newborn) was done differently in NHFS-4 in 2015–2016 and NFHS-5 in 2019–2021 because a different set of variables was given in each data and thus these components lack comparability between the two time periods.

**Figure 2:**
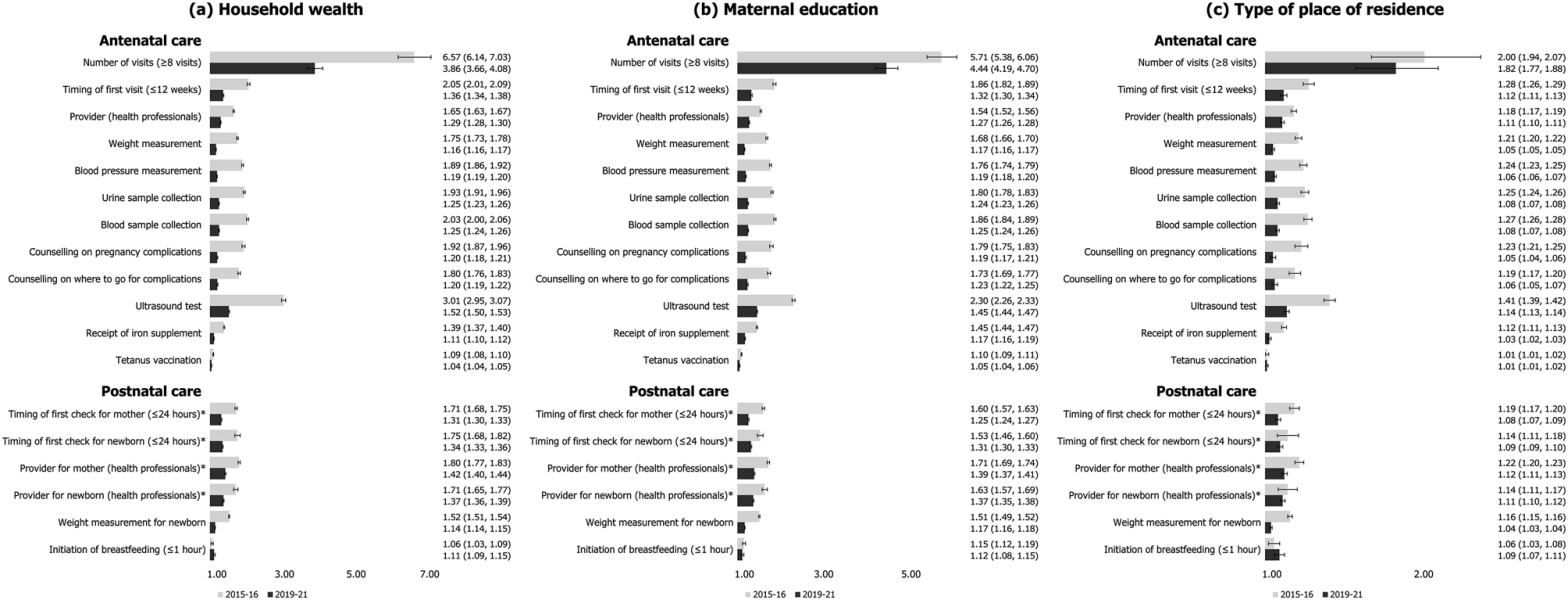
Change in relative inequalities in the prevalence of antenatal and postnatal care components stratified by socioeconomic and geographic predictors, 2015–16 (N = 161,225) vs. 2019–21 (N = 150,611). * The operationalization of four postnatal care components (timing of first check for mother and newborn, provider for mother and newborn) was done differently in NHFS-4 in 2015–16 and NFHS-5 in 2019–21 because a different set of variables was given in each data and thus these components lack comparability between the two time periods.

In 2021, mothers from the richest households consistently showed higher prevalence of receiving each component of ANC/PNC, when compared to mothers from the poorest households (Figs. 1a, 2a). The prevalence of making eight or more ANC visits among the richest mothers was, for example, 23.02%p higher and 3.86 times greater than that among the poorest. The greatest inequality in absolute scale was found for ultrasound test (AI = 33.30%p) and the smallest for tetanus vaccination (AI = 3.80%p). The use of relative scale showed that the prevalence of eight or more ANC visits (RI = 3.86) had greatest inequality by household wealth while that of tetanus vaccination (RI = 1.04) was the smallest.

In general, inequalities in ANC/PNC components by wealth have decreased over time (Figs. 1a, 2a; Supplementary Tables S2 and S3 online). In terms of ultrasound test, the wealth gap between mothers from the richest and poorest quintiles decreased from 64.07%p in 2016 to 33.30%p in 2021. The relative inequality in ANC visits also showed large decrease from 6.57 in 2016 to 3.86 in 2021. There were a few components with escalated inequalities by household wealth over time: timing of first check for newborn (AI = 12.45%p to 22.35%p), provider for newborn (AI = 15.28%p to 25.02%p), and initiation of breastfeeding (AI = 2.42%p to 4.63%p) in absolute term. Only initiation of breastfeeding showed increase in inequality by household wealth in relative scale (RI = 1.06 to 1.11).

#### Education

By and large a similar pattern was found for educational inequalities in ANC/PNC components in 2021 (Figures 1b, 2b). For instance, the absolute and relative inequality between mothers with no education versus highest education was largest for ultrasound test (AI = 30.26%p) and completion of eight or more ANC visits (RI = 4.44), respectively. Inequality by education was found to be smallest for tetanus vaccination in both scales (AI = 4.45%p, RI = 1.05).

Absolute and relative inequalities by education decreased from 2016 to 2021 for most ANC/PNC components (Figures 1b, 2b; Supplementary Tables S2 and S3 online). The decrease over time was greatest for blood pressure measurement in absolute terms (AI = 39.88%p to 15.60%p), and for number of ANC visits in relative terms (RI = 5.71 to 4.44). Postnatal checkups for newborn (AI = 9.68%p to 20.44%p for timing of first check, AI = 14.45%p to 24.53%p for provider) still had an inverse change in inequalities by education.

#### Type of place of residence

Although the size of absolute and relative inequalities by place of residence was comparatively small, mothers in urban areas had higher prevalence of experiencing each component of ANC/PNC compared to those in rural areas (Figs. 1c, 2c). Mothers in urban areas, for example, had 13.22%p higher and 1.82 times greater prevalence of making eight or more ANC visits, and these were the largest inequalities found across all ANC/PNC components. As it was with household wealth and education, absolute and relative inequalities were smallest for tetanus vaccination (AI = 1.07%p, RI = 1.01).

While residential inequalities in both scales declined for most ANC/PNC components over time, the decline was greatest for ultrasound test (AI = 25.00%p to 11.33%p, RI = 1.41 to 1.14) (Figs. 1c, 2c; Supplementary Tables S2 and S3 online). Absolute inequalities by place of residence worsened over time for timely checkup for newborn (AI = 3.21%p to 7.19%p), provider for newborn (AI = 4.28%p to 8.81%p), and initiation of breastfeeding (AI = 2.41%p to 3.87%p). At the relative scale, residential inequality worsened only for initiation of breastfeeding (RI = 1.06 to 1.09).

### State-specific analyses

**Figure 3:**
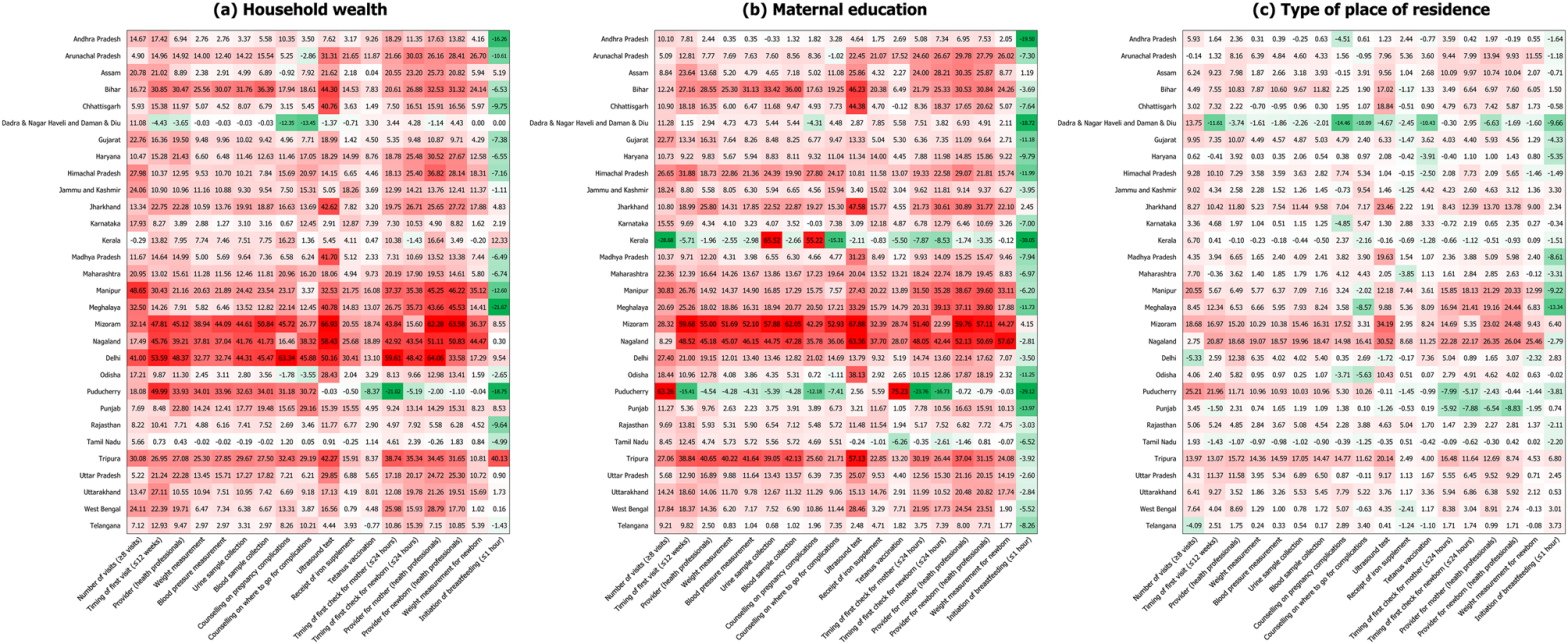
State-specific absolute inequalities in the prevalence of antenatal and postnatal care components stratified by socioeconomic and geographic predictors, 2019–21 (N = 150,611).

**Figure 4:**
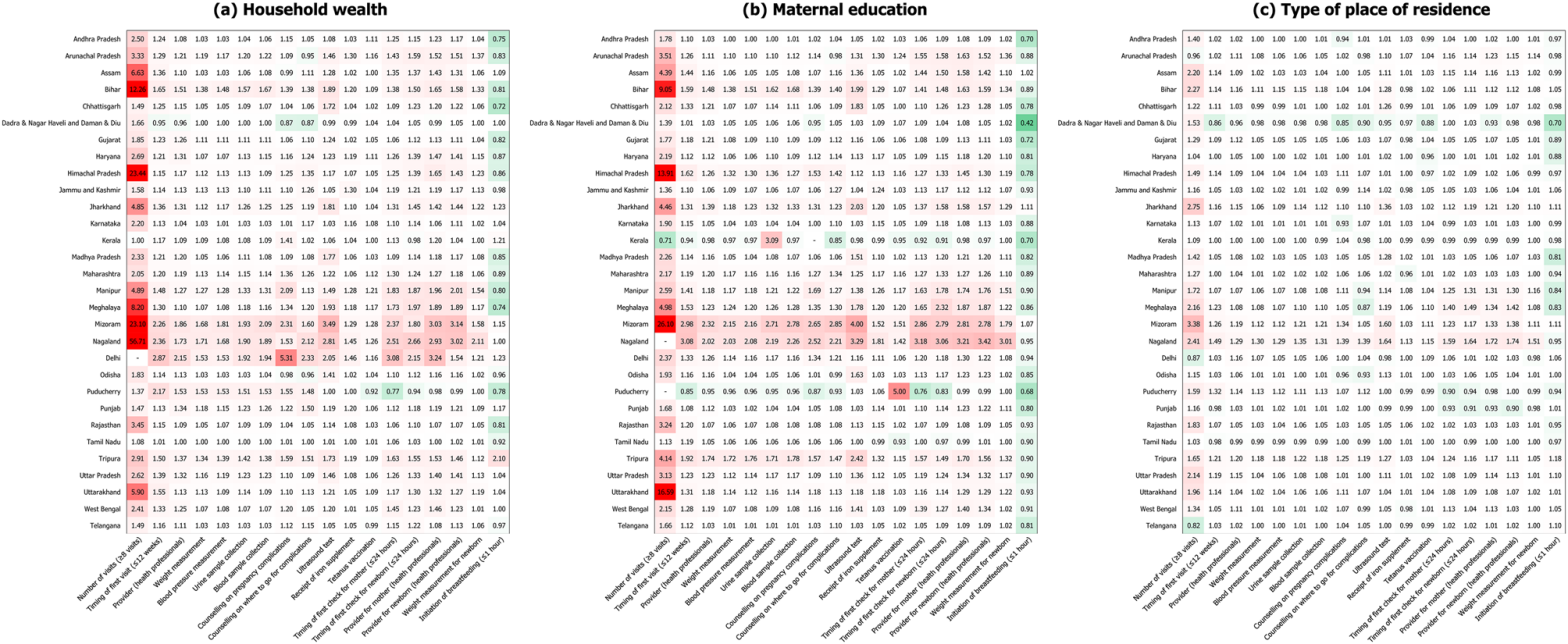
State-specific relative inequalities in the prevalence of antenatal and postnatal care components stratified by socioeconomic and geographic predictors, 2019–21 (N = 150,611). Note. A cell with a hyphen (-) indicates there were no observations in the reference group (e.g., the poorest quintile) who received the corresponding component, and thus it was unable to calculate relative inequality.

When stratified by states, most states showed substantial socioeconomic and geographic inequalities in ANC/PNC components, at both absolute (Fig. 3) and relative scale (Fig. 4). For all three socioeconomic and geographic predictors, Bihar, Mizoram, Nagaland and Tripura had the largest inequalities in most of the components. Wealth inequality in these states, for example, was found to be greatest for ultrasound test in absolute terms (ranging from AI = 66.93%p in Mizoram to AI = 42.27%p in Tripura) and number of ANC visits in relative terms (ranging from RI = 56.71 in Nagaland to RI = 2.91 in Tripura). For more than half of the states, inequalities in the initiation of breastfeeding showed a reversed pattern—that is, mothers from the poorest quintile, with no education, and residing in rural areas had higher prevalence of initiating breastfeeding within an hour of delivery compared to their counterparts.

### Multivariable logistic regression models

**Figure 5:**
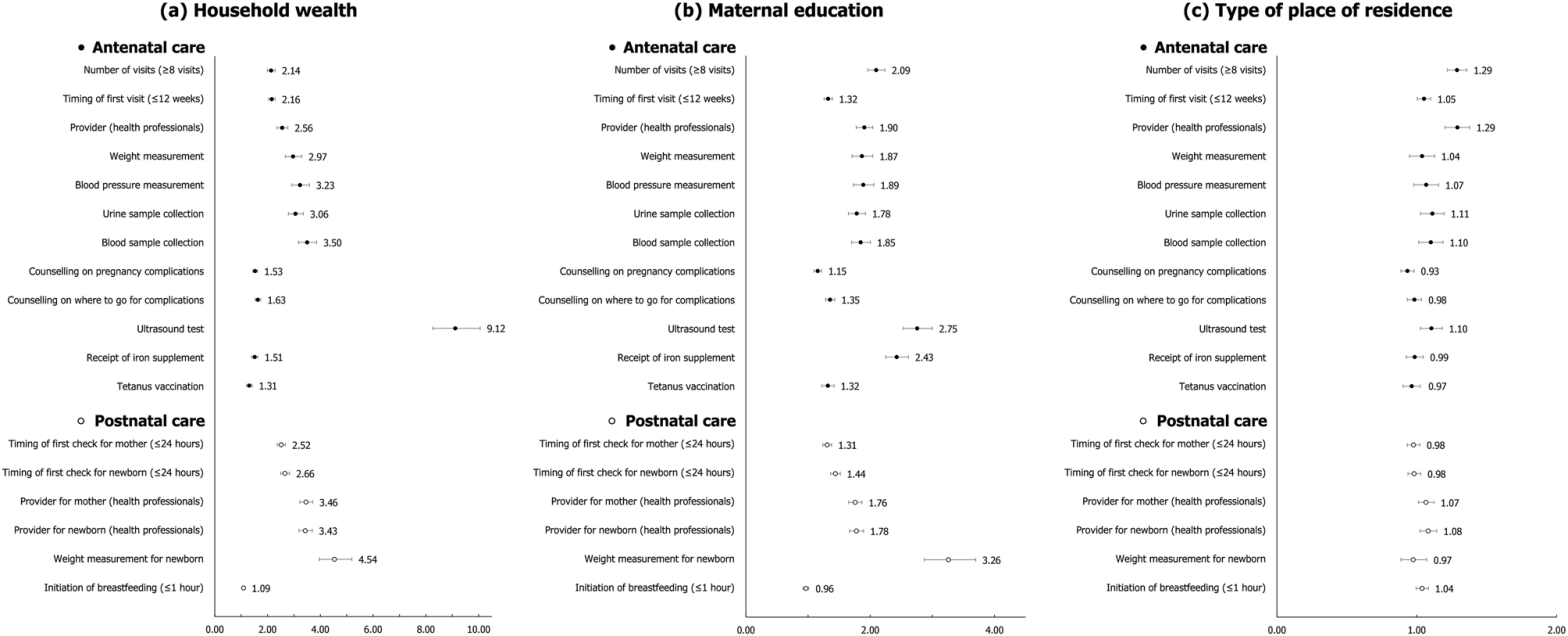
Adjusted odds ratios of the associations between socioeconomic and geographic predictors and antenatal and postnatal care components, 2019–21 (N = 150,611). Logistic regressions adjusted for maternal age at childbirth, sex and birth order of newborn, maternal marital status, caste/tribe, and religion. All models adjusted for clustering of primary sampling units to attain robust standard errors.

We found consistent social gradient in ANC/PNC components even after adjusting for additional sociodemographic features (Fig. 5, Supplementary Table S4). For example, mothers from the richest quintile had greater likelihood of receiving an ultrasound test as part of their ANC (OR = 9.12, 95% CI 8.27–10.06) and having their newborn weighed after delivery (OR = 4.54, 95% CI 3.96–5.20) compared to those from the poorest quintile. There was a similar pattern for education, indicating that mothers with highest education level were more likely to use ANC/PNC components in comparison to those with no education. Initiation of breastfeeding was the only component for which education was not statistically significant (OR = 0.96, 95% CI 0.92–1.01).

On the other hand, type of place of residence showed statistically insignificant associations for half of ANC components, including weight measurement for mother, blood pressure measurement, counselling, receipt of iron supplement, and tetanus vaccination. In terms of PNC components, whether health professionals performed postnatal check for mother (OR = 1.07, 95% CI 1.01–1.12) and newborn (OR = 1.08, 95% CI 1.02–1.14) was associated with type of place of residence at statistically significant level.

## Discussion

From our analysis on socioeconomic and geographic inequalities in the comprehensive utilization of antenatal and postnatal care based on the latest nationally representative survey of young and middle-aged women in India, four salient findings emerged. First, the prevalence of women who received each component of ANC/PNC showed an overall increase between 2016 and 2021. However, despite the overall progress, the prevalence of mothers who made a minimum of eight ANC visits was still low in 2021 (19.8%) and did not show any improvement since 2016 (0.7%-point decrease). One possible explanation for the stagnation can be the difference in the guidelines provided by WHO and the Government of India concerning the minimum number of ANC visits^6,26^. While WHO recommendation increased the number of minimum ANC visits for healthy pregnancy from four to eight times in 2016, the guideline published by the Ministry of Health and Welfare in India remained outdated since 2010^6,26^. Hence, health professionals and pregnant mothers may find ANC visits exceeding four times unnecessary even when it is affordable. Nonetheless, even the prevalence of mothers with four or more ANC visits remains relatively low and stable (52.4% in 2016 versus 59.9% in 2021; data not shown), indicating that efforts are needed to encourage more frequent ANC checkups.

In 2021, several ANC components showed near universal uptake; approximately nine out of ten mothers received physical diagnoses including measurement of weight (91.6%) and blood pressure (90.8%), and collection of urine (88.4%) and blood sample (88.9%) during their pregnancy. These ANC services are essential as they enable health professionals to identify maternal status related to over- and undernutrition, anemia, hypertension, asymptomatic bacteriuria and diabetes, and promote prevention as well as early initiation of treatment to optimize health outcomes of mothers and newborns^6^. Similarly, preventive measures such as receipt of iron supplement (88.2%) and tetanus vaccination (91.1%) had high completion rate in 2021, which are also important components to prevent maternal anemia, puerperal sepsis, preterm birth and neonatal mortality from tetanus^6^.

Second, as expected, mothers and their newborns from poorer households, with lower education level, and residing in rural areas were less likely to use ANC/PNC components on both absolute and relative scale. Our finding aligns with prior studies reporting that maternal SES and their place of residence are associated with uptake of perinatal care in India^8–14^. To this extensive literature, our study adds that the magnitude of inequality in ANC/PNC was greatest by household wealth quintiles and smallest by urban versus rural residence, and that these inequalities have reduced over time between 2016 and 2021.

The implementation of the Pradhan Mantri Surakshit Matritva Abhiyan (PMSMA) launched by the Ministry of Health and Family Welfare, Government of India may be one of the potential drivers that improved the utilization of perinatal care while reducing socioeconomic inequalities in tandem. Aimed to provide assured, comprehensive ANC universally to all pregnant women at free of cost, the PMSMA has been offering a package of physical diagnoses and essential nutritional supplements as part of ANC since 2016^1,30^. This could have disproportionately benefited the less privileged mothers, thereby reducing socioeconomic inequalities over time.

Third, the extent of social gradient in ANC/PNC differed by specific components. For example, whether a mother received an ultrasound test showed greatest absolute inequality according to her SES and residence. Ultrasound test helps estimate gestational age, detect fetal anomalies and reduce labor time of post-term pregnancy, and hence improves the overall quality of pregnancy experience^6^. The statistically significant social gradient observed in the use of ultrasound test may be attributed to the fact that ultrasound test requires more financial resources than other ANC components. The place of ANC utilization can also matter as a previous study from rural Kenya has shown that mothers who used ANC from government hospitals, health centers and dispensaries were less likely to receive an ultrasound test compared to those whose ANC service was provided in private/mission facilities^31^. Although the PMSMA is providing a free ultrasound test during the second trimester of pregnancy, health facilities in public sectors may not be well-equipped enough to provide the service in reality^30,31^. It is thus important to ensure health facilities with adequate training and equipment specific to ultrasound test to properly deliver such services across all social groups.

On the other hand, early initiation of breastfeeding showed only a small to null association with maternal SES and residence, both at the national- and state-level. Initiation of breastfeeding within the first hour of birth is a highly recommended PNC since it can improve a newborn’s immune response and help prevent infections in a cost-effective manner^32,33^. Prior studies have reported mixed results of the association between household wealth and early initiation of breastfeeding in LMICs^32,34–37^. While mothers from poorer households tend to rely more on breastfeeding due to domestic food insecurity, their limited use of ANC and more frequent home delivery may lead to less promotion of breastfeeding at the same time^32,35–37^. Similarly, richer mothers in LMICs may be more exposed to media promotion of breast milk substitutes, leading to decreased likelihood of breastfeeding^37^. Conversely, maternal education was consistently reported to have a positive association with timely initiation of breastfeeding since mothers with higher education level had greater awareness about breastfeeding and better response to health messages given by health professionals^34–36^.

Lastly, socioeconomic inequalities in the uptake of perinatal care varied across states, with the largest disparity found in the northeastern India including Bihar, Mizoram, Nagaland and Tripura. These states are landlocked, physically separated from the mainland with insufficient means of transportation, and thus may have benefited less from the healthcare improvement that has been achieved in the southern part of the country^38^. The overall development such as economic growth, human development and infrastructure facilities in the northeastern part of India also remains poor, which may exacerbate the social disparity within the use of maternity care^39^. The tribal distribution may have also affected the larger inequalities observed in the northeastern states wherein most population belongs to aboriginal tribes and are more likely to rely on traditional practices rather than modern healthcare practices^38,40^. Building on the ongoing policy interventions of the government of India, additional efforts to prioritize these underperforming social groups are essential to successfully address inequalities within various ANC/PNC components in these states^41^.

In contrast, comparatively small inequalities were observed across states in the southern region including Karnataka, Kerala and Tamil Nadu. In addition to their high level of economic development, a various set of maternal health care schemes have been reported to minimize socioeconomic disparities in the use of maternity care. For example, Tamil Nadu runs the conditional cash transfer scheme (i.e., Dr Muthulakshmi Reddy Maternity Benefit Scheme) with the highest amount of beneficiary payment in India (₹18,000)^42^. Pregnant mothers and newborns in Tamil Nadu are also tracked from the early stage of pregnancy so that their use of whole perinatal care services including ANC, birth registration and immunization can be monitored and evaluated at the state level^42^. These services, along with the efforts of the central government of India such as Janani Suraksha Yojana (cash incentives for institutional deliveries), PMSMA and Ayushman Bharat-Pradhan Mantri Jan Arogya Yojana (health insurance scheme for catastrophic expenditure on healthcare) may have had synergistic effects to successfully reduce socioeconomic inequalities in the use of perinatal care in these states^1^.

We acknowledge a few important data and methodological limitations. Though NFHS provides a diverse set of variables on mothers and newborns’ utilization on perinatal care, some essential components recommended by the national and international guidelines (e.g., assessment of tobacco/substance use during pregnancy, carrying a case note, number of PNC visits, counselling on more specific topics) were not collected in the data, hence limiting our analysis to the selected components^6,25^. Second, caste/tribe is another important social characteristic in the context of India. However, current literature suggests inconsistent associations between caste/tribe identity and utilization of perinatal care,^8–12,43^ and in our logistic regression models—where caste/tribe was adjusted for—the magnitude and statistical significance of their associations with the outcomes were relatively weak compared to those of our main predictors. Therefore, this study did not present an in-depth examination of the role of caste/tribe groups, but future studies should examine this aspect to benefit the existing knowledge.

Third, as the fieldwork for the latest NFHS was conducted between June 2019 and April 2021, the outbreak of COVID-19 may have affected the ANC/PNC use due to disruptions in health services^44–46^. For example, restricted public transportation and lockdown, fear of infection in health facilities, and mistrust in health systems could have limited mothers’ access to seek for essential perinatal care during the pandemic^45,46^. Likewise, the overburden of healthcare workers and facilities to cope with the patients from COVID-19 could have restricted their capacity for timely and adequate provision of perinatal care^46^. However, a recent study in India reported that most child health outcomes including use of ANC remained generally stable before and after the outbreak^44,47^, although the long-term effects of the pandemic need further assessment. Lastly, responses used to construct ANC/PNC components in our analysis were solely based on mother’s recall about their latest childbirth and were not validated from medical records.

Despite these limitations, our study provides comprehensive evidence in the recent progress that has been made in the use of ANC/PNC in India and which specific components require further improvement. For example, the number of ANC visits and ultrasound test were found to be relatively low on average and even more restricted for low SES groups and rural residents. We also found that although the extent of socioeconomic and geographic inequalities has reduced, there remains substantial gap by wealth and education groups, especially in northeastern states. To maximize the government’s ongoing efforts to offer free-of-cost ANC services and facility delivery, it is essential to raise awareness of women and their partners about the importance of perinatal care and advance health infrastructures particularly in less developed regions of India. Future interventions addressing the quality of perinatal care in India should aim to achieve universal coverage as well as the comprehensive utilization of all essential ANC/PNC components to improve maternal and newborn health.

### Supplementary Information

Supplementary Information.

## Data Availability

This project used publicly accessible secondary data obtained from the Demographic and Health Surveys (DHS) website. The DHS data were not collected specifically for this study and no one on the study team has access to identifiers linked to the data. These activities do not meet the regulatory definition of human subject research. As such, an Institutional Review Board (IRB) review is not required. HC and RK had full access to all the data in the study and takes responsibility for the integrity of the data and the accuracy of the data analysis.
