# Supplementary material for: Socioeconomic and geographic inequalities in antenatal and postnatal care components in India, 2016–2021
Source: Sci Rep. 2024 May 3;14:10221. doi: 10.1038/s41598-024-59981-w (PMC11068794; doi:10.1038/s41598-024-59981-w)
Supplement: Supplementary file 1 — Supplementary Information. [file 41598_2024_59981_MOESM1_ESM.docx]

**Socioeconomic and geographic inequalities in antenatal and postnatal care components in India, 2016-2021**

Hyejun Chi, Sohee Jung, S.V. Subramanian, Rockli Kim

**SUPPLEMENTARY MATERIALS**

**Supplementary Figure S1a. Flow chart of sample construction, 2019-21 (N=150,611)**


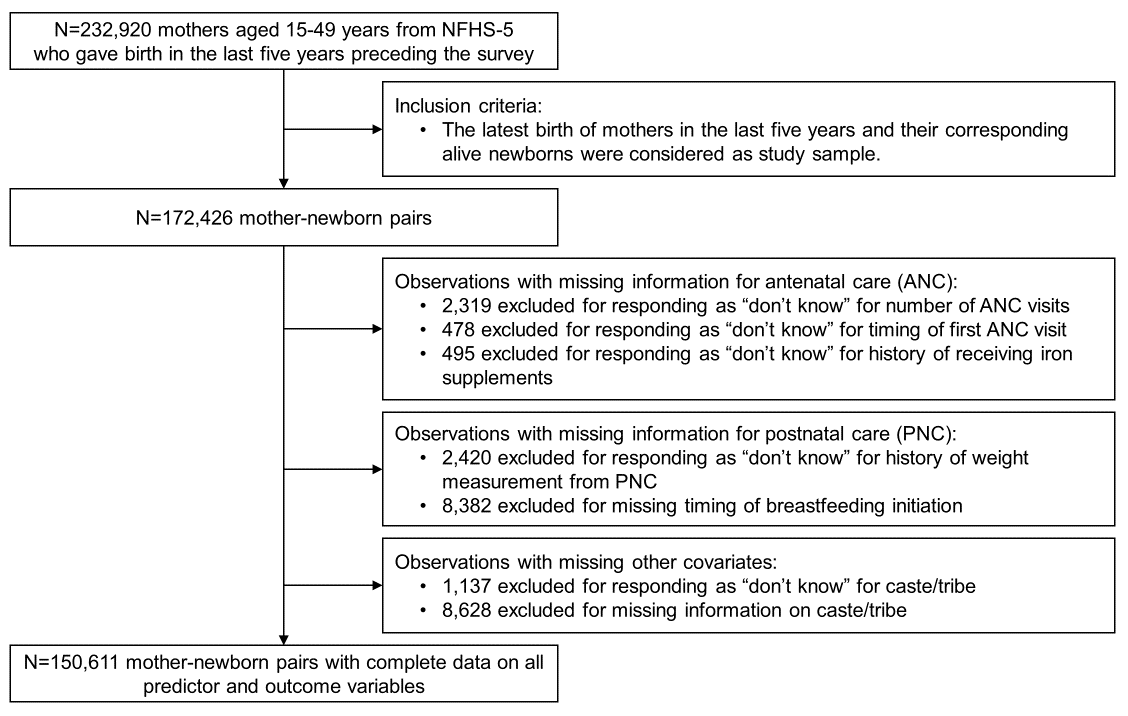


Note. NFHS-5: National Family and Health Survey Round-5 from 2019-21.

**Supplementary Figure S1b. Flow chart of sample construction, 2015-16 (N=161,225)**


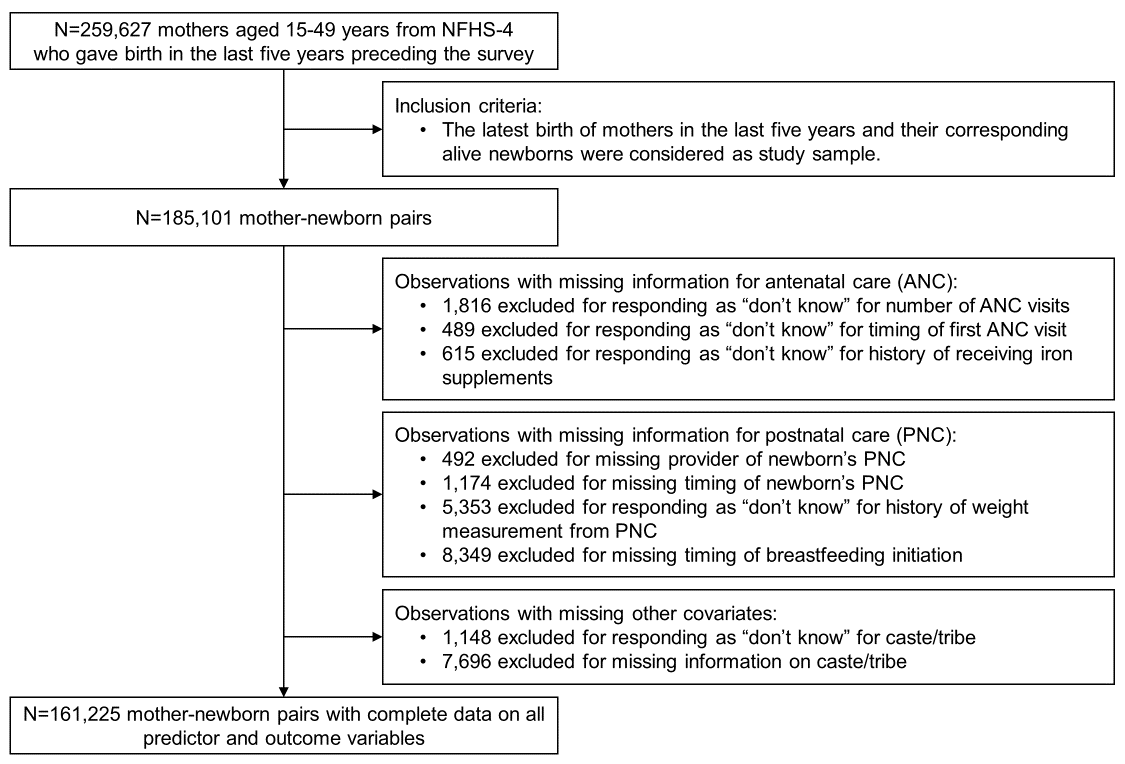


Note. NFHS-4: National Family and Health Survey Round-4 from 2015-16.

**Supplementary Table S1. Descriptive of sociodemographic characteristics of the study sample, 2015-16 (N=161,225) vs. 2019-21 (N=150,611)**

|  | 2015-16  (N=161,225) | 2019-21  (N=150,611) |
| --- | --- | --- |
| **Socioeconomic and geographic predictors** | N (%) | |
| Household wealth |  |  |
| Poorest | 39,402 (24.4%) | 37,269 (24.7%) |
| Poorer | 36,189 (22.4%) | 34,100 (22.6%) |
| Middle | 32,169 (20.0%) | 29,717 (19.7%) |
| Richer | 28,298 (17.6%) | 26,843 (17.8%) |
| Richest | 25,167 (15.6%) | 22,682 (15.1%) |
| Education |  |  |
| No education | 45,288 (28.1%) | 30,074 (20.0%) |
| Primary | 22,421 (13.9%) | 18,153 (12.1%) |
| Secondary | 75,711 (47.0%) | 78,829 (52.3%) |
| Higher than secondary | 17,805 (11.0%) | 23,555 (15.6%) |
| Type of place of residence |  |  |
| Rural | 120,247 (74.6%) | 118,239 (78.5%) |
| Urban | 40,978 (25.4%) | 32,372 (21.5%) |
| **Other sociodemographic characteristics** | N (%) | |
| Maternal age at birth (years) |  |  |
| < 20 | 15,818 (9.8%) | 13,184 (8.8%) |
| 20-24 | 67,303 (41.7%) | 61,111 (40.6%) |
| 25-29 | 49,589 (30.8%) | 48,982 (32.5%) |
| 30-34 | 19,859 (12.3%) | 19,800 (13.1%) |
| 35-39 | 6,685 (4.1%) | 6,030 (4.0%) |
| 40-44 | 1,739 (1.1%) | 1,334 (0.9%) |
| 45-49 | 232 (0.1%) | 170 (0.1%) |
| Sex of newborn |  |  |
| Boy | 87,532 (54.3%) | 80,867 (53.7%) |
| Girl | 73,693 (45.7%) | 69,744 (46.3%) |
| Birth order of newborn |  |  |
| 1 | 52,379 (32.5%) | 50,683 (33.7%) |
| 2-3 | 81,250 (50.4%) | 79,060 (52.5%) |
| 4+ | 27,595 (17.1%) | 20,868 (13.9%) |
| Maternal marital status |  |  |
| Never in a union | 263 (0.2%) | 216 (0.1%) |
| Currently married/living with a partner | 158,545 (98.3%) | 148,228 (98.4%) |
| Widowed/divorced/separated | 2,418 (1.5%) | 2,167 (1.4%) |
| Caste |  |  |
| Belong to a scheduled caste/tribe/other backward class | 130,318 (80.8%) | 124,303 (82.5%) |
| None of them | 30,907 (19.2%) | 26,308 (17.5%) |
| Religion |  |  |
| Hindu | 121,063 (75.1%) | 115,220 (76.5%) |
| Muslim | 20,034 (12.4%) | 17,190 (11.4%) |
| Christian | 13,167 (8.2%) | 12,020 (8.0%) |
| Other/no religion | 6,961 (4.3%) | 6,181 (4.1%) |

**Supplementary Table S2a. Socioeconomic and geographic absolute inequalities (%-point) in the national prevalence of antenatal and postnatal care components, 2019-21 (N=150,611)**

|  | **Absolute inequality* (95% confidence interval)** | | |
| --- | --- | --- | --- |
| **Socioeconomic and geographic predictors** | **Household wealth** | **Education** | **Place of residence** |
| **Antenatal care** |  |  |  |
| Number of visits (≥8 visits) | 23.02 (22.40, 23.65) | 24.99 (24.34, 25.63) | 13.22 (12.78, 13.67) |
| Timing of first visit (≤12 weeks) | 21.30 (20.58, 22.02) | 19.17 (18.43, 19.92) | 8.07 (7.56, 8.57) |
| Provider (health professionals) | 21.21 (20.66, 21.75) | 19.88 (19.32, 20.45) | 9.01 (8.62, 9.40) |
| Weight measurement | 13.66 (13.22, 14.10) | 13.65 (13.19, 14.10) | 4.55 (4.24, 4.86) |
| Blood pressure measurement | 15.69 (15.23, 16.14) | 15.60 (15.13, 16.07) | 5.45 (5.13, 5.78) |
| Urine sample collection | 18.77 (18.27, 19.28) | 18.51 (17.99, 19.03) | 6.84 (6.48, 7.20) |
| Blood sample collection | 19.09 (18.60, 19.58) | 18.88 (18.38, 19.39) | 6.82 (6.47, 7.17) |
| Counselling on pregnancy complications | 12.93 (12.22, 13.64) | 12.22 (11.49, 12.95) | 3.57 (3.07, 4.07) |
| Counselling on where to go for complications | 13.85 (13.17, 14.53) | 15.44 (14.74, 16.14) | 4.56 (4.08, 5.04) |
| Ultrasound test | 33.30 (32.77, 33.82) | 30.26 (29.71, 30.81) | 11.33 (10.95, 11.72) |
| Receipt of iron supplement | 9.06 (8.54, 9.57) | 13.92 (13.39, 14.45) | 2.41 (2.05, 2.77) |
| Tetanus vaccination | 3.80 (3.34, 4.26) | 4.45 (3.98, 4.93) | 1.07 (0.75, 1.39) |
| **Postnatal care** |  |  |  |
| Timing of first check for mother (≤24 hours) | 20.49 (19.83, 21.16) | 16.92 (16.23, 17.61) | 6.14 (5.66, 6.61) |
| Timing of first check for newborn (≤24 hours) | 22.35 (21.69, 23.01) | 20.44 (19.77, 21.12) | 7.19 (6.72, 7.65) |
| Provider for mother (health professionals) | 27.10 (26.47, 27.72) | 25.07 (24.43, 25.72) | 9.40 (8.95, 9.84) |
| Provider for newborn (health professionals) | 25.02 (24.42, 25.62) | 24.53 (23.91, 25.15) | 8.81 (8.39, 9.24) |
| Weight measurement for newborn | 12.23 (11.86, 12.61) | 14.31 (13.93, 14.70) | 3.47 (3.20, 3.73) |
| Initiation of breastfeeding (≤1 hour) | 4.63 (3.83, 5.43) | 4.52 (3.70, 5.34) | 3.87 (3.32, 4.43) |

* For absolute inequality, we subtracted the prevalence of the comparing group (the richest quintile for household wealth, higher than secondary education, urban residence) from that of the reference group (the poorest quintile, no education, rural residence). Differences were weighted using mother’s sampling weight divided by 1,000,000 and presented in percentage points.

**Supplementary Table S2b. Socioeconomic and geographic absolute inequalities (%-point) in the national prevalence of antenatal and postnatal care components, 2015-16 (N=161,225)**

|  | **Absolute inequality* (95% confidence interval)** | | |
| --- | --- | --- | --- |
| **Socioeconomic and geographic predictors** | **Household wealth** | **Education** | **Place of residence** |
| **Antenatal care** |  |  |  |
| Number of visits (≥8 visits) | 31.84 (31.23, 32.44) | 32.28 (31.63, 32.94) | 15.86 (15.43, 16.28) |
| Timing of first visit (≤12 weeks) | 39.96 (39.23, 40.70) | 35.52 (34.73, 36.32) | 15.12 (14.60, 15.64) |
| Provider (health professionals) | 37.01 (36.42, 37.60) | 32.92 (32.28, 33.56) | 13.70 (13.28, 14.12) |
| Weight measurement | 39.75 (39.13, 40.38) | 37.10 (36.43, 37.77) | 15.08 (14.63, 15.53) |
| Blood pressure measurement | 43.89 (43.26, 44.51) | 39.88 (39.21, 40.56) | 16.91 (16.46, 17.36) |
| Urine sample collection | 44.52 (43.88, 45.15) | 40.65 (39.96, 41.34) | 17.26 (16.80, 17.72) |
| Blood sample collection | 46.88 (46.25, 47.52) | 42.47 (41.78, 43.16) | 18.36 (17.90, 18.83) |
| Counselling on pregnancy complications | 32.60 (31.84, 33.36) | 29.24 (28.43, 30.05) | 11.55 (11.02, 12.07) |
| Counselling on where to go for complications | 31.46 (30.71, 32.21) | 29.71 (28.91, 30.52) | 10.16 (9.63, 10.68) |
| Ultrasound test | 64.07 (63.45, 64.69) | 53.22 (52.52, 53.92) | 25.00 (24.52, 25.48) |
| Receipt of iron supplement | 25.02 (24.40, 25.64) | 28.51 (27.85, 29.17) | 9.05 (8.62, 9.48) |
| Tetanus vaccination | 7.82 (7.33, 8.32) | 7.97 (7.44, 8.50) | 1.12 (0.78, 1.47) |
| **Postnatal care** |  |  |  |
| Timing of first check for mother (≤24 hours) | 30.81 (30.07, 31.56) | 27.68 (26.88, 28.48) | 10.49 (9.97, 11.01) |
| Timing of first check for newborn (≤24 hours) | 12.45 (11.79, 13.11) | 9.68 (8.97, 10.39) | 3.21 (2.76, 3.67) |
| Provider for mother (health professionals) | 36.22 (35.51, 36.93) | 34.38 (33.62, 35.15) | 13.31 (12.81, 13.82) |
| Provider for newborn (health professionals) | 15.28 (14.56, 16.00) | 14.45 (13.68, 15.22) | 4.28 (3.79, 4.78) |
| Weight measurement for newborn | 33.29 (32.74, 33.84) | 32.53 (31.94, 33.11) | 12.49 (12.10, 12.89) |
| Initiation of breastfeeding (≤1 hour) | 2.42 (1.65, 3.19) | 5.77 (4.94, 6.60) | 2.41 (1.88, 2.94) |

* For absolute inequality, we subtracted the prevalence of the comparing group (the richest quintile for household wealth, higher than secondary education, urban residence) from that of the reference group (the poorest quintile, no education, rural residence). Differences were weighted using mother’s sampling weight divided by 1,000,000 and presented in percentage points.

**Supplementary table S3a. Socioeconomic and geographic relative inequalities in the national prevalence of antenatal and postnatal care components, 2019-21 (N=150,611)**

|  | **Relative inequality* (95% confidence interval)** | | |
| --- | --- | --- | --- |
| **Socioeconomic and geographic predictors** | **Household wealth** | **Education** | **Place of residence** |
| **Antenatal care** |  |  |  |
| Number of visits (≥8 visits) | 3.86 (3.66, 4.08) | 4.44 (4.19, 4.70) | 1.82 (1.77, 1.88) |
| Timing of first visit (≤12 weeks) | 1.36 (1.34, 1.38) | 1.32 (1.30, 1.34) | 1.12 (1.11, 1.13) |
| Provider (health professionals) | 1.29 (1.28, 1.30) | 1.27 (1.26, 1.28) | 1.11 (1.10, 1.11) |
| Weight measurement | 1.16 (1.16, 1.17) | 1.17 (1.16, 1.17) | 1.05 (1.05, 1.05) |
| Blood pressure measurement | 1.19 (1.19, 1.20) | 1.19 (1.18, 1.20) | 1.06 (1.06, 1.07) |
| Urine sample collection | 1.25 (1.23, 1.26) | 1.24 (1.23, 1.26) | 1.08 (1.07, 1.08) |
| Blood sample collection | 1.25 (1.24, 1.26) | 1.25 (1.24, 1.26) | 1.08 (1.07, 1.08) |
| Counselling on pregnancy complications | 1.20 (1.18, 1.21) | 1.19 (1.17, 1.21) | 1.05 (1.04, 1.06) |
| Counselling on where to go for complications | 1.20 (1.19, 1.22) | 1.23 (1.22, 1.25) | 1.06 (1.05, 1.07) |
| Ultrasound test | 1.52 (1.50, 1.53) | 1.45 (1.44, 1.47) | 1.14 (1.13, 1.14) |
| Receipt of iron supplement | 1.11 (1.10, 1.12) | 1.17 (1.16, 1.19) | 1.03 (1.02, 1.03) |
| Tetanus vaccination | 1.04 (1.04, 1.05) | 1.05 (1.04, 1.06) | 1.01 (1.01, 1.02) |
| **Postnatal care** |  |  |  |
| Timing of first check for mother (≤24 hours) | 1.31 (1.30, 1.33) | 1.25 (1.24, 1.27) | 1.08 (1.07, 1.09) |
| Timing of first check for newborn (≤24 hours) | 1.34 (1.33, 1.36) | 1.31 (1.30, 1.33) | 1.09 (1.09, 1.10) |
| Provider for mother (health professionals) | 1.42 (1.40, 1.44) | 1.39 (1.37, 1.41) | 1.12 (1.11, 1.13) |
| Provider for newborn (health professionals) | 1.37 (1.36, 1.39) | 1.37 (1.35, 1.38) | 1.11 (1.10, 1.12) |
| Weight measurement for newborn | 1.14 (1.14, 1.15) | 1.17 (1.16, 1.18) | 1.04 (1.03, 1.04) |
| Initiation of breastfeeding (≤1 hour) | 1.11 (1.09, 1.15) | 1.12 (1.08, 1.15) | 1.09 (1.07, 1.11) |

* For relative inequality by socioeconomic and geographic predictors, we divided the prevalence of the comparing group (the richest quintile for household wealth, higher than secondary education, urban residence) by that of the reference group (the poorest quintile, no education, rural residence). Differences were weighted using mother’s sampling weight divided by 1,000,000.

**Supplementary table S3b. Socioeconomic and geographic relative inequalities in the national prevalence of antenatal and postnatal care components, 2015-16 (N=161,225)**

|  | **Relative inequality* (95% confidence interval)** | | |
| --- | --- | --- | --- |
| **Socioeconomic and geographic predictors** | **Household wealth** | **Education** | **Place of residence** |
| **Antenatal care** |  |  |  |
| Number of visits (≥8 visits) | 6.57 (6.14, 7.03) | 5.71 (5.38, 6.06) | 2.00 (1.94, 2.07) |
| Timing of first visit (≤12 weeks) | 2.05 (2.01, 2.09) | 1.86 (1.82, 1.89) | 1.28 (1.26, 1.29) |
| Provider (health professionals) | 1.65 (1.63, 1.67) | 1.54 (1.52, 1.56) | 1.18 (1.17, 1.19) |
| Weight measurement | 1.75 (1.73, 1.78) | 1.68 (1.66, 1.70) | 1.21 (1.20, 1.22) |
| Blood pressure measurement | 1.89 (1.86, 1.92) | 1.76 (1.74, 1.79) | 1.24 (1.23, 1.25) |
| Urine sample collection | 1.93 (1.91, 1.96) | 1.80 (1.78, 1.83) | 1.25 (1.24, 1.26) |
| Blood sample collection | 2.03 (2.00, 2.06) | 1.86 (1.84, 1.89) | 1.27 (1.26, 1.28) |
| Counselling on pregnancy complications | 1.92 (1.87, 1.96) | 1.79 (1.75, 1.83) | 1.23 (1.21, 1.25) |
| Counselling on where to go for complications | 1.80 (1.76, 1.83) | 1.73 (1.69, 1.77) | 1.19 (1.17, 1.20) |
| Ultrasound test | 3.01 (2.95, 3.07) | 2.30 (2.26, 2.33) | 1.41 (1.39, 1.42) |
| Receipt of iron supplement | 1.39 (1.37, 1.40) | 1.45 (1.44, 1.47) | 1.12 (1.11, 1.13) |
| Tetanus vaccination | 1.09 (1.08, 1.10) | 1.10 (1.09, 1.11) | 1.01 (1.01, 1.02) |
| **Postnatal care** |  |  |  |
| Timing of first check for mother (≤24 hours) | 1.71 (1.68, 1.75) | 1.60 (1.57, 1.63) | 1.19 (1.17, 1.20) |
| Timing of first check for newborn (≤24 hours) | 1.75 (1.68, 1.82) | 1.53 (1.46, 1.60) | 1.14 (1.11, 1.18) |
| Provider for mother (health professionals) | 1.80 (1.77, 1.83) | 1.71 (1.69, 1.74) | 1.22 (1.20, 1.23) |
| Provider for newborn (health professionals) | 1.71 (1.65, 1.77) | 1.63 (1.57, 1.69) | 1.14 (1.11, 1.17) |
| Weight measurement for newborn | 1.52 (1.51, 1.54) | 1.51 (1.49, 1.52) | 1.16 (1.15, 1.16) |
| Initiation of breastfeeding (≤1 hour) | 1.06 (1.03, 1.09) | 1.15 (1.12, 1.19) | 1.06 (1.03, 1.08) |

* For relative inequality by socioeconomic and geographic predictors, we divided the prevalence of the comparing group (the richest quintile for household wealth, higher than secondary education, urban residence) by that of the reference group (the poorest quintile, no education, rural residence). Differences were weighted using mother’s sampling weight divided by 1,000,000.

**Supplementary table S4a. Associations between household wealth and antenatal and postnatal care components, 2019-21 (N=150,611)**

|  | **Adjusted odds ratio by household wealth (95% confidence interval)** | | | |
| --- | --- | --- | --- | --- |
|  | **Poorer** | **Middle** | **Richer** | **Richest** |
| **Antenatal care** |  |  |  |  |
| Number of visits (≥8 visits) | 1.42 (1.35, 1.51) | 1.85 (1.74, 1.96) | 2.19 (2.06, 2.33) | 2.14 (1.99, 2.29) |
| Timing of first visit (≤12 weeks) | 1.21 (1.17, 1.25) | 1.54 (1.48, 1.60) | 1.86 (1.78, 1.95) | 2.16 (2.04, 2.29) |
| Provider (health professionals) | 1.56 (1.49, 1.63) | 2.08 (1.97, 2.19) | 2.44 (2.29, 2.60) | 2.56 (2.36, 2.77) |
| Weight measurement | 1.50 (1.42, 1.58) | 2.07 (1.93, 2.21) | 2.62 (2.41, 2.85) | 2.97 (2.67, 3.30) |
| Blood pressure measurement | 1.54 (1.46, 1.62) | 2.13 (2.00, 2.28) | 2.69 (2.49, 2.92) | 3.23 (2.91, 3.59) |
| Urine sample collection | 1.54 (1.47, 1.62) | 2.13 (2.01, 2.26) | 2.61 (2.43, 2.81) | 3.06 (2.79, 3.36) |
| Blood sample collection | 1.54 (1.47, 1.62) | 2.21 (2.08, 2.35) | 2.87 (2.66, 3.09) | 3.50 (3.18, 3.86) |
| Counselling on pregnancy complications | 1.13 (1.08, 1.17) | 1.28 (1.22, 1.34) | 1.32 (1.26, 1.39) | 1.53 (1.44, 1.63) |
| Counselling on where to go for complications | 1.13 (1.09, 1.17) | 1.34 (1.28, 1.40) | 1.45 (1.38, 1.53) | 1.63 (1.53, 1.74) |
| Ultrasound test | 2.25 (2.17, 2.35) | 4.19 (3.98, 4.42) | 6.73 (6.28, 7.22) | 9.12 (8.27, 10.06) |
| Receipt of iron supplement | 1.14 (1.09, 1.20) | 1.29 (1.22, 1.36) | 1.36 (1.27, 1.45) | 1.51 (1.40, 1.64) |
| Tetanus vaccination | 1.14 (1.09, 1.21) | 1.15 (1.08, 1.21) | 1.21 (1.14, 1.30) | 1.31 (1.21, 1.42) |
| **Postnatal care** |  |  |  |  |
| Timing of first check for mother (≤24 hours) | 1.34 (1.29, 1.38) | 1.65 (1.58, 1.72) | 1.96 (1.87, 2.06) | 2.52 (2.37, 2.68) |
| Timing of first check for newborn (≤24 hours) | 1.40 (1.35, 1.45) | 1.75 (1.68, 1.83) | 2.06 (1.97, 2.17) | 2.66 (2.50, 2.83) |
| Provider for mother (health professionals) | 1.46 (1.41, 1.52) | 2.07 (1.98, 2.17) | 2.69 (2.55, 2.84) | 3.46 (3.23, 3.71) |
| Provider for newborn (health professionals) | 1.49 (1.44, 1.55) | 2.09 (1.99, 2.19) | 2.64 (2.49, 2.79) | 3.43 (3.19, 3.69) |
| Weight measurement for newborn | 1.72 (1.62, 1.82) | 2.67 (2.48, 2.88) | 3.62 (3.28, 3.99) | 4.54 (3.96, 5.20) |
| Initiation of breastfeeding (≤1 hour) | 1.05 (1.01, 1.09) | 1.08 (1.03, 1.12) | 1.12 (1.07, 1.17) | 1.09 (1.03, 1.15) |

Note. Logistic regressions adjusted for education, type of place of residence, maternal age at childbirth, sex and birth order of newborn, maternal marital status, caste/tribe, and religion. All models adjusted for clustering of primary sampling units to attain robust standard errors. Poorest household wealth served as the reference category.

**Supplementary table S4b. Association between education and antenatal and postnatal care components, 2019-21 (N=150,611)**

|  | **Adjusted odds ratio by education (95% confidence interval)** | | |
| --- | --- | --- | --- |
|  | **Primary** | **Secondary** | **Higher than secondary** |
| **Antenatal care** |  |  |  |
| Number of visits (≥8 visits) | 1.21 (1.14, 1.30) | 1.80 (1.70, 1.90) | 2.09 (1.96, 2.24) |
| Timing of first visit (≤12 weeks) | 1.15 (1.10, 1.20) | 1.29 (1.25, 1.34) | 1.32 (1.26, 1.39) |
| Provider (health professionals) | 1.34 (1.28, 1.41) | 1.64 (1.57, 1.71) | 1.90 (1.77, 2.04) |
| Weight measurement | 1.45 (1.37, 1.55) | 1.81 (1.72, 1.91) | 1.87 (1.71, 2.04) |
| Blood pressure measurement | 1.45 (1.37, 1.54) | 1.86 (1.76, 1.96) | 1.89 (1.73, 2.06) |
| Urine sample collection | 1.45 (1.37, 1.53) | 1.81 (1.73, 1.90) | 1.78 (1.65, 1.92) |
| Blood sample collection | 1.48 (1.41, 1.57) | 1.86 (1.77, 1.95) | 1.85 (1.70, 2.00) |
| Counselling on pregnancy complications | 1.17 (1.12, 1.23) | 1.27 (1.22, 1.32) | 1.15 (1.09, 1.22) |
| Counselling on where to go for complications | 1.27 (1.22, 1.33) | 1.37 (1.32, 1.43) | 1.35 (1.28, 1.43) |
| Ultrasound test | 1.35 (1.29, 1.42) | 1.74 (1.67, 1.81) | 2.75 (2.53, 3.00) |
| Receipt of iron supplement | 1.39 (1.31, 1.46) | 1.82 (1.74, 1.90) | 2.43 (2.25, 2.61) |
| Tetanus vaccination | 1.19 (1.12, 1.27) | 1.27 (1.21, 1.34) | 1.32 (1.22, 1.42) |
| **Postnatal care** |  |  |  |
| Timing of first check for mother (≤24 hours) | 1.19 (1.14, 1.24) | 1.37 (1.32, 1.42) | 1.31 (1.24, 1.38) |
| Timing of first check for newborn (≤24 hours) | 1.26 (1.21, 1.32) | 1.43 (1.38, 1.49) | 1.44 (1.36, 1.52) |
| Provider for mother (health professionals) | 1.30 (1.25, 1.36) | 1.62 (1.56, 1.69) | 1.76 (1.65, 1.87) |
| Provider for newborn (health professionals) | 1.30 (1.24, 1.36) | 1.63 (1.57, 1.69) | 1.78 (1.67, 1.89) |
| Weight measurement for newborn | 1.54 (1.44, 1.64) | 2.14 (2.02, 2.27) | 3.26 (2.87, 3.70) |
| Initiation of breastfeeding (≤1 hour) | 1.09 (1.05, 1.13) | 1.12 (1.08, 1.16) | 0.96 (0.92, 1.01) |

Note. Logistic regressions adjusted for household wealth, type of place of residence, maternal age at childbirth, sex and birth order of newborn, maternal marital status, caste/tribe, and religion. All models adjusted for clustering of primary sampling units to attain robust standard errors. No education served as the reference category.

**Supplementary table S4c. Association between type of place of residence and antenatal and postnatal care components, 2019-21 (N=150,611)**

|  | **Adjusted odds ratio by type of place of residence**  **(95% confidence interval)** |
| --- | --- |
|  | **Urban** |
| **Antenatal care** |  |
| Number of visits (≥8 visits) | 1.29 (1.22, 1.36) |
| Timing of first visit (≤12 weeks) | 1.05 (1.01, 1.10) |
| Provider (health professionals) | 1.29 (1.21, 1.38) |
| Weight measurement | 1.04 (0.95, 1.13) |
| Blood pressure measurement | 1.07 (0.99, 1.16) |
| Urine sample collection | 1.11 (1.03, 1.20) |
| Blood sample collection | 1.10 (1.02, 1.19) |
| Counselling on pregnancy complications | 0.93 (0.89, 0.98) |
| Counselling on where to go for complications | 0.98 (0.93, 1.03) |
| Ultrasound test | 1.10 (1.03, 1.18) |
| Receipt of iron supplement | 0.99 (0.93, 1.05) |
| Tetanus vaccination | 0.97 (0.92, 1.03) |
| **Postnatal care** |  |
| Timing of first check for mother (≤24 hours) | 0.98 (0.93, 1.02) |
| Timing of first check for newborn (≤24 hours) | 0.98 (0.94, 1.03) |
| Provider for mother (health professionals) | 1.07 (1.01, 1.12) |
| Provider for newborn (health professionals) | 1.08 (1.02, 1.14) |
| Weight measurement for newborn | 0.97 (0.89, 1.07) |
| Initiation of breastfeeding (≤1 hour) | 1.04 (0.99, 1.08) |

Note. Logistic regressions adjusted for household wealth, education, maternal age at childbirth, sex and birth order of newborn, maternal marital status, caste/tribe, and religion. All models adjusted for clustering of primary sampling units to attain robust standard errors. Rural residence served as the reference category.
